# *Gastrodia elata* Ameliorates High-Fructose Diet-Induced Lipid Metabolism and Endothelial Dysfunction

**DOI:** 10.1155/2014/101624

**Published:** 2014-02-26

**Authors:** Min Chul Kho, Yun Jung Lee, Jeong Dan Cha, Kyung Min Choi, Dae Gill Kang, Ho Sub Lee

**Affiliations:** ^1^College of Oriental Medicine and Professional Graduate School of Oriental Medicine, Wonkwang University, Shinyong-dong, Iksan, Jeonbuk 570-749, Republic of Korea; ^2^Hanbang Body-Fluid Research Center, Wonkwang University, Shinyong-dong, Iksan, Jeonbuk 570-749, Republic of Korea; ^3^Department of Research Development, Institute of Jinan Red Ginseng, Jinan, Jeonbuk 567-801, Republic of Korea

## Abstract

Overconsumption of fructose results in dyslipidemia, hypertension, and impaired glucose tolerance, which have documented correlation with metabolic syndrome. *Gastrodia elata*, a widely used traditional herbal medicine, was reported with anti-inflammatory and antidiabetes activities. Thus, this study examined whether ethanol extract of *Gastrodia elata* Blume (EGB) attenuate lipid metabolism and endothelial dysfunction in a high-fructose (HF) diet animal model. Rats were fed the 65% HF diet with/without EGB 100 mg/kg/day for 8 weeks. Treatment with EGB significantly suppressed the increments of epididymal fat weight, blood pressure, plasma triglyceride, total cholesterol levels, and oral glucose tolerance, respectively. In addition, EGB markedly prevented increase of adipocyte size and hepatic accumulation of triglycerides. EGB ameliorated endothelial dysfunction by downregulation of endothelin-1 (ET-1) and adhesion molecules in the aorta. Moreover, EGB significantly recovered the impairment of vasorelaxation to acetylcholine and levels of endothelial nitric oxide synthase (eNOS) expression and induced markedly upregulation of phosphorylation AMP-activated protein kinase (AMPK)**α** in the liver, muscle, and fat. These results indicate that EGB ameliorates dyslipidemia, hypertension, and insulin resistance as well as impaired vascular endothelial function in HF diet rats. Taken together, EGB may be a beneficial therapeutic approach for metabolic syndrome.

## 1. Introduction

Metabolic syndrome, a worldwide issue, is characterized by insulin resistance, impaired glucose tolerance and/or hyperglycemia, high blood serum triglycerides, low concentration of high-density lipoprotein (HDL) cholesterol, high blood pressure, and central obesity. The association of 3 (or more) of these factors leads to an increased morbidity and mortality from several predominant diseases such as type 2 diabetes, cancer, and cardiovascular diseases including atherosclerosis, myocardial infarction, and stroke [1, 2].

Fructose is an isomer of glucose with a hydroxyl group on carbon-4 reversed in position. It is promptly absorbed and rapidly metabolized by liver. Recent decades westernization of diets has resulted in significant increases in added fructose, enormous rised in fructose consumption typical daily [3]. The exposure of the liver to such enormous rising fructose consumption leads to rapid stimulation of lipogenesis and triglyceride accumulation, which in turn leads to reduced insulin sensitivity and hepatic insulin resistance/glucose intolerance [4]. Thus, high-fructose diet induces a well-characterised metabolic syndrome, generally resulting in hypertension, dyslipidaemia, and low level of HDL-cholesterol [5]. Recent studies suggest that high fructose intake may be an important risk factor for the development of fatty liver [6]. Rats are commonly used as a model to mimic human disease, including metabolic syndrome [7]. Similarly, emerging data suggest that experiment on fructose-diet rats tends to produce some of the changes associated with metabolic syndrome, such as altered lipid metabolism, fatty liver, hypertension, obesity, and dyslipidemia [8].

*Gastrodia elata* Blume is a traditional herbal medicine in Korea, China, and Japan, which has been used for the treatment of headaches, hypertension, rheumatism, and cardiovascular diseases [9]. Several major physiological substances have been identified from *Gastrodia elata* Blume such as gastrodin, vanillyl alcohol, vanillin, glycoprotein, p-endoxybenzyl alcohol, and polysaccharides including alpha-D-glucan [10–12]. Our previous studies showed that *Gastrodia elata* Blume exhibits anti-inflammatory and antiatherosclerotic properties by inhibiting the expression of proinflammatory cytokines in vascular endothelial cells [13, 14]. However, the effect of ethanol extract of *Gastrodia elata* Blume on high-fructose (HF) diet animal model has not been yet reported. Thus, the present study was designed to determine whether an ethanol extract of *Gastrodia elata* Blume (EGB) improves high-fructose diet-induced lipid metabolism and endothelial dysfunction.

## 2. Materials and Methods

### 2.1. Preparation of *Gastrodia elata* Blume

The *Gastrodia elata *Blume was purchased from the Herbal Medicine Co-operative Association, Iksan, Jeonbuk Province, Korea, in May 2012. A voucher specimen (no. HBJ1041) was deposited in the herbarium of the Professional Graduate School of Oriental Medicine, Wonkwang University, Iksan, Jeonbuk, South Korea. The dried *Gastrodia elata *Blume (400 g) was extracted with 4 L of 95% ethanol at room temperature for 1 week. The extract was filtered through Whatman no. 3 filter paper (Whatman International Ltd., England) and concentrated using rotary evaporator. The resulting extract (12.741 g) was lyophilized by using a freeze drier and retained until required.

### 2.2. Animal Experiments and Diet

All experimental procedures were carried out in accordance with the National Institute of Health Guide for the Care and Use of Laboratory Animals and were approved by the Institutional Animal Care and Utilization Committee for Medical Science of Wonkwang University. Seven week-old male Sprague-Dawley (SD) rats were obtained from Samtako (Osan, Korea). Rats were kept in a room automatically maintained at a temperature (23 ± 2°C), humidity (50~60%), and 12-h light/dark cycle throughout the experiments. After 1 week of acclimatization, animals were randomly divided into three groups (*n* = 10 per group). Control group (Cont.) was fed regular diet, high-fructose group (HF) was fed 65% fructose diet (Research Diet, USA), and the third group (HF + EGB) was fed with 65% fructose along with a single dose of 100 mg/kg/day of EGB orally for a period of 8 weeks. The regular diet was composed of 50% starch, 21% protein, 4% fat, and standard vitamins and mineral mix. The high-fructose diet was composed of 65% fructose, 20% protein, 10% fat, and standard vitamins and mineral mix.

### 2.3. Blood and Tissue Sampling

At the end of the experiments, the aorta, liver, adipose tissue (epididymal fat pads), and muscle were separated and frozen until analysis after being rinsed with cold saline. The plasma was obtained from the coagulated blood by centrifugation at 3,000 rpm 15 min at 4°C. The separation of plasma was frozen at −80°C until analysis.

### 2.4. Measurements of Blood Pressure

Systolic blood pressure (SBP) was determined by using noninvasive tail-cuff plethysmography method and recorded with an automatic sphygmotonography (MK2000; Muromachi Kikai, Tokyo, Japan). The systolic blood pressure (SBP) was measured at week 1, week 3, and week 7, respectively. At least seven determinations were made in every session. Values were presented as the mean ± SEM of five measurements.

### 2.5. Analysis of Plasma Lipids

The levels of triglyceride in plasma were measured by using commercial kits (ARKRAY, Inc., MINAMI-KU, KYOTO, Japan). The levels of high-density lipoprotein (HDL)-cholesterol, total cholesterol, and LDL-cholesterol in plasma were measured by using HDL and LDL assay kit (E2HL-100, BioAssay Systems).

### 2.6. Estimation of Blood Glucose and Oral Glucose Tolerance Test

The concentration of glucose in blood was measured which was obtained from tail vein using glucometer (Onetouch Ultra) and Test Strip (Life Scan Inc., CA, USA), respectively.

The oral glucose tolerance test (OGTT) was performed 2 days apart at 7 weeks. For the OGTT, briefly, basal blood glucose concentrations were measured after 10~12 h of overnight food privation; then the glucose solution (2 g/kg body weight) was immediately administered via oral gavage, and fourth more tail vein blood samples were taken at 30, 60, 90, and 120 min after glucose administration.

### 2.7. Preparation of Carotid Artery and Measurement of Vascular Reactivity

The carotid arteries of the rats were rapidly and carefully isolated and placed into cold Kreb's solution of the following composition (mM): NaCl 118, KCl 4.7, MgSO_4_ 1.1, KH_2_PO_4_ 1.2, CaCl 1.5, NaHCO_3_ 25, glucose 10, and pH 7.4. The carotid arteries were removed to connective tissue and fat and cut into rings of approximately 3 mm in length. All dissecting procedures were carried out for caring to protect the endothelium from accidental damage. The carotid artery rings were suspended by means of two L-shaped stainless-steel wires inserted into the lumen in a tissue bath containing Kreb's solution at 37°C and aerated with 95% O_2_ and 5% CO_2_. The isometric forces of the rings were measured by using a Grass FT 03 force displacement transducer connected to a Model 7E polygraph recording system (Grass Technologies, Quincy, MA, USA). In the carotid artery rings of rats, a passive stretch of 1 g was determined to be optimal tension for maximal responsiveness to phenylephrine (10^−6^ M). The preparations were allowed to equilibrate for approximately 1 h with an exchange of Kreb's solution every 10 min. The relaxant effects of acetylcholine (ACh, 10^−9^~10^−6^ M) and sodium nitroprusside (SNP, 10^−10^~10^−5^ M) were studied in carotid artery rings constricted submaximally with phenylephrine (10^−6^ M).

### 2.8. Western Blot Analysis in the Rat Aorta, Liver, Muscle, and Fat

The aorta, liver muscle, and fat tissues homogenate were prepared in ice-cold buffer containing 250 mM sucrose, 1 mM EDTA, 0.1 mM phenylmethylsufonyl fluoride, and 20 mM potassium phosphate buffer (pH 7.6). The homogenates were then centrifuged at 8,000 rpm for 10 min at 4°C, and the supernatant was centrifuged at 13,000 rpm for 5 min at 4°C, and as a cytosolic fraction for the analysis of protein. The recovered proteins were separated by 10% SDS-polyacrylamide gel electrophoresis and electrophoresis transferred to nitrocellulose membranes. Membranes were blocked by 5% BSA powder in 0.05% Tween 20-Tris-bufferd saline (TBS-T) for 1 h. The antibodies against ICAM-1, VCAM-1, E-selectin, eNOS, ET-1 (in aorta), AMPK, and p-AMPK (in liver, muscle, and fat) were purchased from Santa Cruz Biotechnology, Inc. (Santa Cruz, CA, USA). The nitrocellulose membranes were incubated overnight at 4°C with protein antibodies. The blots were washed several times with TBS-T and incubated with horseradish peroxidase-conjugated secondary antibody for 1 h, and then the immunoreactive bands were visualized by using enhanced chemiluminescence (Amersham, Buchinghamshire, UK). The bands were analyzed densitometrically by using a Chemi-doc image analyzer (Bio-Rad, Hercules, CA, USA).

### 2.9. Histopathological Staining of Aorta, Epididymal Fat, and Liver

Aortic tissues were fixed in 10% (v/v) formalin in 0.01 M phosphate buffered saline (PBS) for 2 days with change of formalin solution every day to remove traces of blood from tissue. The tissue samples were dehydrated and embedded in paraffin, and then thin sections (6 *μ*m) of the aortic arch in each group were cut and stained with hematoxylin and eosin (H&E). Epididymal fat and liver tissues were fixed by immersion in 4% paraformaldehyde for 48 h at 4°C and incubated with 30% sucrose for 2 days. Each fat and liver was embedded in OCT compound (Tissue-Tek, Sakura Finetek, Torrance, CA, USA), frozen in liquid nitrogen, and stored at −80°C. Frozen sections were cut with a Shandon Cryotome SME (Thermo Electron Corporation, Pittsburg, PA, USA) and placed on poly-L-lysine-coated slide. Epididymal fat sections were stained with H&E. Liver sections were assessed by using Oil Red O staining. For quantitative histopathological comparisons, each section was determined by Axiovision 4 Imaging/Archiving software.

### 2.10. Immunihistochemical Staining of Aortic Tissues

Paraffin sections for immunohistochemical staining were placed on poly-L-lysine-coated slide (Fisher scientific, Pittsburgh, PA, USA). Slides were immunostained by Invitrogen's HISOTO-STAIN-SP kits using the Labeled-(strept) Avidin-Biotin (LAB-SA) method. After antigen retrieval, slides were immersed in 3% hydrogen peroxide for 10 min at room temperature to block endogenous peroxidase activity and rinsed with PBS. After being rinsed, slides were incubated with 10% nonimmune goat serum for 10 min at room temperature and incubated with primary antibodies of ICAM-1, VCAM-1, and E-selectin (1:200; Santa Cruz, CA, USA) in humidified chambers overnight at 4°C. All slides were then incubated with biotinylated secondary antibody for 20 min at room temperature and then incubated with horseradish peroxidase-conjugated streptavidin for 20 min at room temperature. Peroxidase activity was visualized by 3,3′-Diaminobenzidine (DAB; Novex, CA) substrate-chromogen system, counterstaining with hematoxylin (Zymed, CA, USA). For quantitative analysis, the average score of 10~20 randomly selected area was calculated by using NIH Image analysis software, Image J (NIH, Bethesda, MD, USA).

### 2.11. Statistical Analysis

All the experiments were repeated at least three times. The results were expressed as a mean ±SD or mean ±SE. The data was analyzed using SIGMAPLOT 10.0 program. The Student's *t*-test was used to determine any significant differences. *P* < 0.05 was considered as statistically significant.

## 3. Results

### 3.1. Characteristics of Experimental Animals

During the entire experimental period, all groups showed significant increase in body weight. There was no significant change in body weight after 8 weeks of fructose feeding in HF group. However, treatment of EGB group showed significant decrease in body weight (439.8 ± 26.5 versus 402.5 ± 22.1, *P* < 0.05) (Table 1). Moreover, HF diet results in a significant increase in epididymal fat pads weight. The weight of epididymal fat pads was 60.8 ± 17.4% higher than that of the HF diet group compared with control group. However, treatment of EGB group significantly reduced the epididymal fat pads weight (57.5 ± 7.3%) compared with HF diet group (Table 1).

### 3.2. Effect of EGB on Blood Pressure

**Figure 1:**
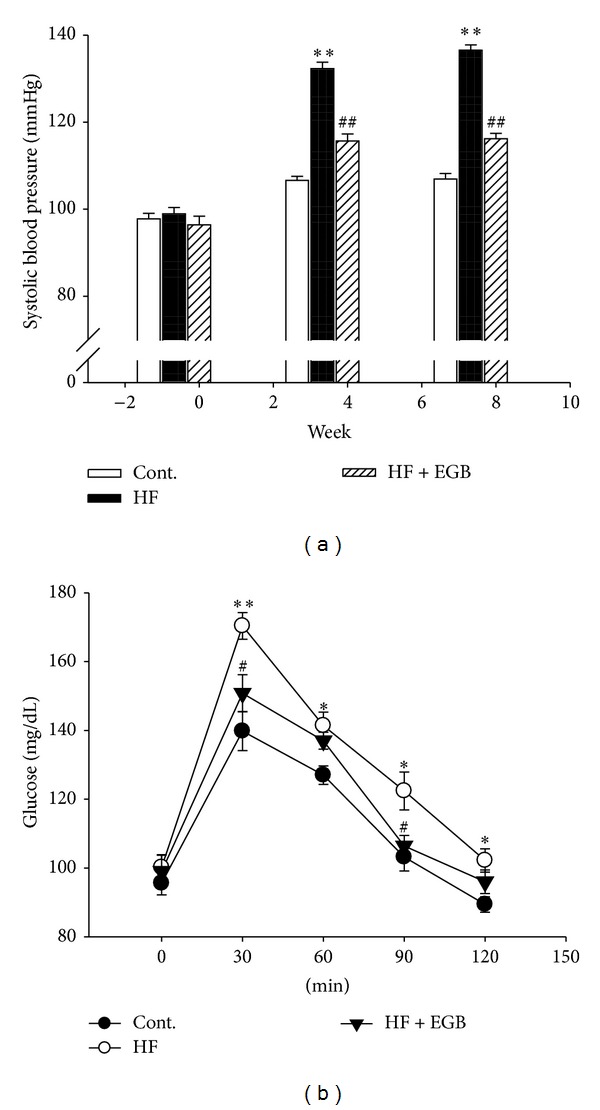
Effects of EGB on systolic blood pressure (a) and oral glucose tolerance test (b). Values were expressed as mean ± SE (*n* = 10). *P < 0.05, **P < 0.01 versus Cont.; ^#^ *P* < 0.05, ^##^ *P* < 0.01 versus HF.

At the beginning of the experimental feeding period, the levels of systolic blood pressure in all groups were approximately 95~100 mmHg as investigated by the tail-cuff technique. After 4 weeks, systolic blood pressure of HF group was significantly increased than that of control group (*P* < 0.01). However, EGB group was significantly decreased than that of HF group during all the experimental period (136.71 ± 1.24 versus 116.4 ± 1.21, *P* < 0.01) (Figure 1(a)).

### 3.3. Effect of EGB on Blood Glucose Level and Oral Glucose Tolerance Test

Plasma blood glucose levels were not statistically different in HF diet rats with chronic treatment of EGB (Table 1). Oral glucose tolerance test was carried out to check insulin resistance in high-fructose diet rats after 8 weeks. The results showed that HF diet group maintained the significant increase in blood glucose levels at 30, 60, 90 (*P* < 0.01), and 120 min (*P* < 0.05), respectively. However, the plasma glucose levels in treatment of EGB were significantly decreased at 30 and 90 min as compared with HF diet group (*P* < 0.05) (Figure 1(b)).

### 3.4. Effect of EGB on Plasma Lipids

Group fed a HF diet displayed was increased plasma triglyceride levels, total cholesterol levels, and LDL-c levels; however, treatment of EGB group significantly decreased plasma triglyceride levels (272.67 ± 107.0 versus 177.33 ± 59.6, *P* < 0.05), total cholesterol levels (102.94 ± 19.7 versus 67.79 ± 5.8, *P* < 0.01), and LDL-c levels (44.56 ± 8.1 versus 24.28 ± 3.1, *P* < 0.01), respectively. Beside the plasma levels of HDL-c levels in EGB group increased compared with HF diet group (16.02 ± 2.9 versus 20.2 ± 2.2, *P* < 0.05) (Table 2).

### 3.5. Effect of EGB on Vascular Tension

**Figure 2:**
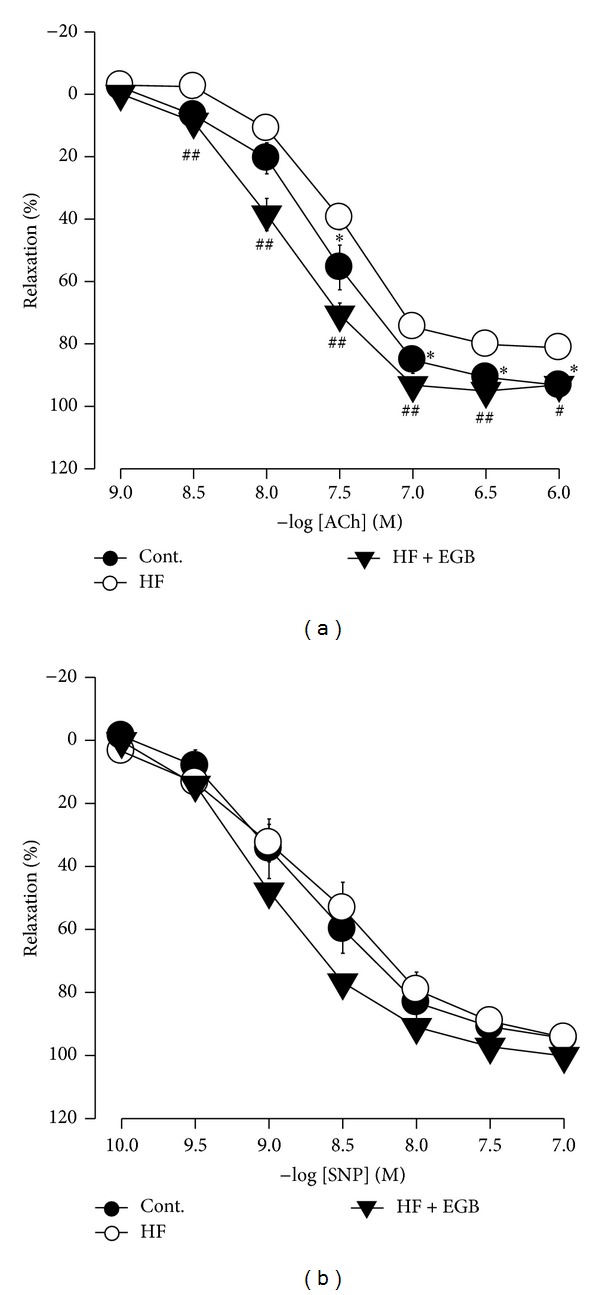
Effect of EGB on relaxation of carotid arteries. Cumulative concentration-response curves to acetylcholine (ACh), endothelium-dependent vasodilator (a) and sodium nitroprusside (SNP), endothelium-independent vasodilator (b) in phenylephrine precontracted carotid arteries from experiment rats. Values were expressed as mean ± SE (*n* = 5). *P < 0.05 versus Cont.; ^#^ *P* < 0.05, ^##^ *P* < 0.01 versus HF.

Vascular responses to ACh, endothelium-dependent vasodilator (1 × 10^−9^ to 1 × 10^−6^ M), SNP, and endothelium-independent vasodilator (1 × 10^−10^ to 1 × 10^−7^ M) were measured in carotid artery. Responses to ACh-induced relaxation of carotid artery rings were significantly decreased in the HF diet group compared with control group (1 × 10^−7.5^ to 1 × 10^−6^ M. *P* < 0.05). However, the impairment of vasorelaxation was remarkably attenuated by treatment with EGB (1 × 10^−8.5^ to 1 × 10^−6.5^ M. *P* < 0.01; 1 × 10^−6^ M. *P* < 0.05) (Figure 2(a)). On the other hand, response to SNP-induced relaxation of carotid artery rings had no significant difference in all the groups (Figure 2(b)).

### 3.6. Effect of EGB on the Morphology of Aorta and Epididymal Fat Pads

**Figure 3:**
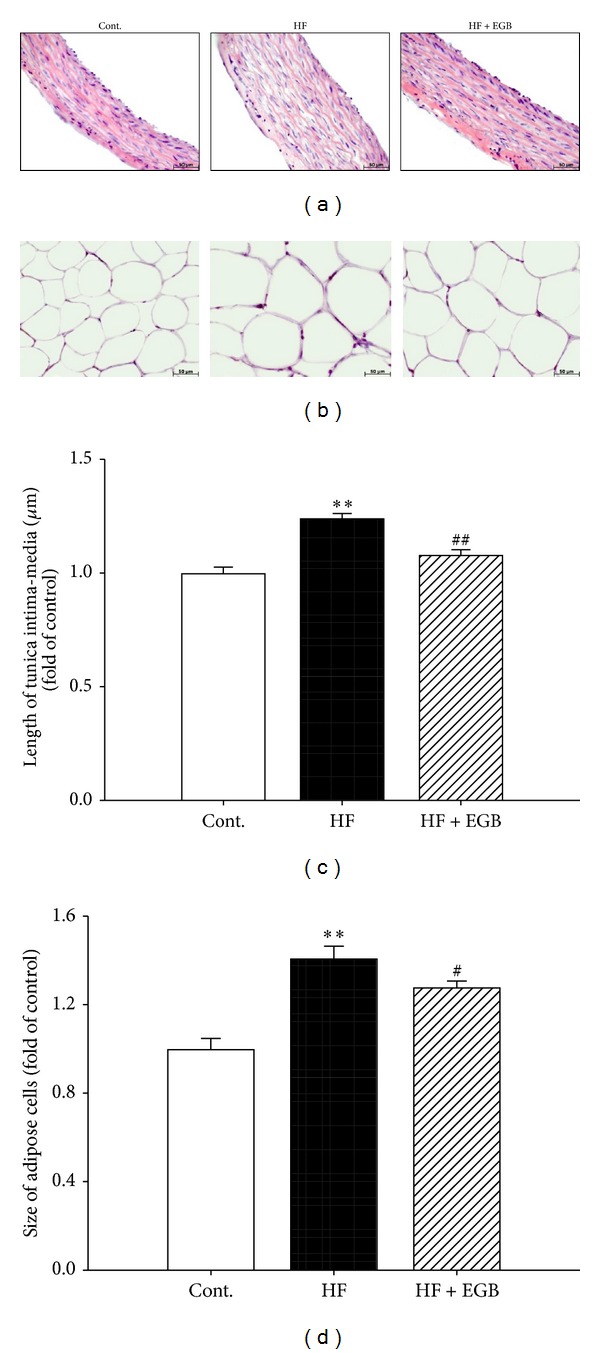
Effects of EGB on aortic wall and adipocytes in HF diet rats. Representative microscopic photographs of H&E stained section of the thoracic aorta (a) and epididymal fat pads (b) in HF diet rats. Lower panel indicated the length of intima-media (c) and size of adipose cells (magnification ×400). Values were expressed as mean ± SE (*n* = 5). **P < 0.01 versus Cont.; ^#^ *P* < 0.05, ^##^ *P* < 0.01 versus HF.

EGB effectively decreased blood pressure and attenuated impairment of vasorelaxation. Thus, we examined histological changes by staining with H&E in thoracic aorta.  Figure 3 showed that thoracic aorta of HF diet group revealed roughened endothelial layers and increased tunica intima-media of layers compared with control group (+24.13%, *P* < 0.01). However, treatment of EGB group significantly maintained the smooth character of the intima endothelial layers and decreased tunica intima-media thickness in aortic section (−16.10%, *P* < 0.01) (Figures 3(a) and 3(c)).

Because EGB effectively reduced the epididymal fat pads weight, we prepared frozen section of epididymal fat pads and stained with H&E. The adipocytes were hypertrophy induced by HF diet compared with control group (+40.97%, *P* < 0.01). However, treatment of EGB significantly decreased the hypertrophy of adipocytes (−13.04%, *P* < 0.05) (Figures 3(b), and 3(d)).

### 3.7. Effect of EGB on the Hepatic Lipids

**Figure 4:**
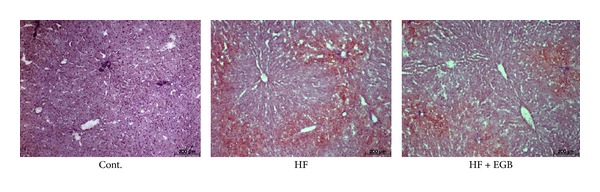
Effect of EGB on fatty liver in HF diet rats. Representative microscopic photographs of Oil Red O stained section of the liver.

To investigate the existence of fat accumulation of liver in all experimental groups, we prepared frozen section of liver and stained with Oil Red O. Lipid droplets were detected in HF diet groups. However, treatment of EGB showed that the number of lipid droplets significantly decreased compared with HF diet group (Figure 4).

### 3.8. Effect of EGB on the Expressions Levels of Adhesion Molecules, eNOS, and ET-1 in Aorta

**Figure 5:**
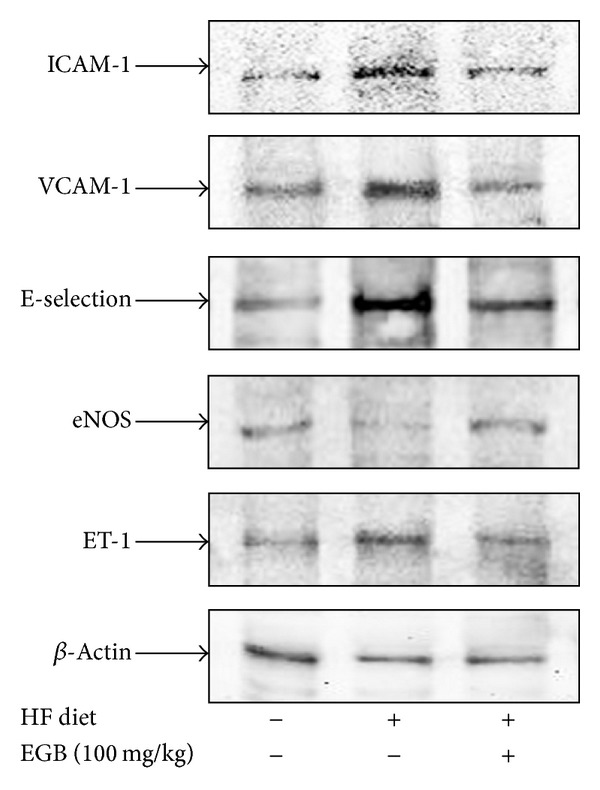
Effects of EGB on the expression of adhesion molecules, eNOS and ET-1 in the aorta of HF diet rats. Each electrophoretogram is representative of the results from three individual experiments.

Protein expression levels of VCAM-1, ICAM-1, E-selectin, eNOS, and ET-1 in aorta were determined by western blotting, respectively. Adhesion molecules (VCAM-1, ICAM-1, and E-selectin) and ET-1 protein levels were increased in the HF diet group compared with control group. However, treatment of EGB group significantly decreased expression levels of protein compared with HF diet group. Moreover, we examined the expression of eNOS levels to evaluate vascular endothelial function. The eNOS protein levels decreased in the HF diet group compared with control group. However, treatment of EGB group increased expression levels of protein compared with HF diet group (Figure 5).

**Figure 6:**
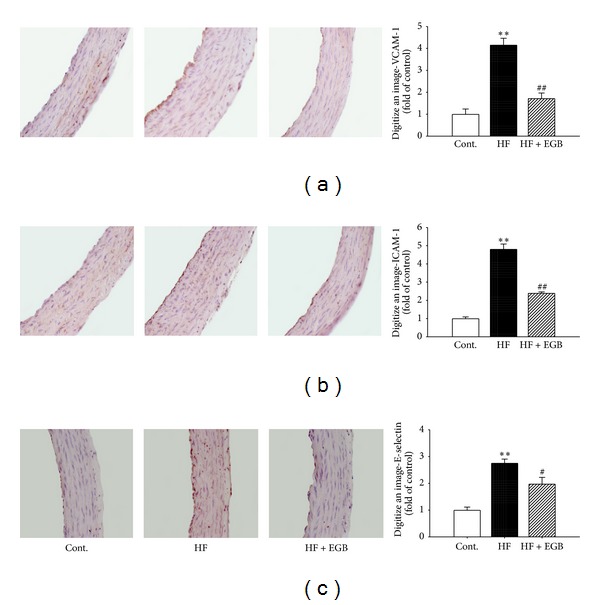
Effects of EGB on VCAM-1 (a), ICAM-1 (b), and E-selectin (c) immunoreactivity in aortic tissues of HF diet rats. Representative immunohistochemistry (left) and quantifications (right) are shown. Values were expressed as mean ± SE **P < 0.01 versus Cont.; ^#^ *P* < 0.05, ^##^ *P* < 0.01 versus HF.

Immunohistochemistry was performed to determine the direct expression of adhesion molecules in the aortic wall. Adhesion molecules expressions such as VCAM-1, ICAM-1, and E-selectin were increased in the HF diet group (*P* < 0.01); however, treatment of EGB group significantly decreased expression levels of protein (VCAM-1, ICAM-1. *P* < 0.01; E-selectin. *P* < 0.05) (Figure 6).

### 3.9. Effect of EGB on the Expressions Levels of AMPK in Liver, Muscle, and Fat Tissues

**Figure 7:**
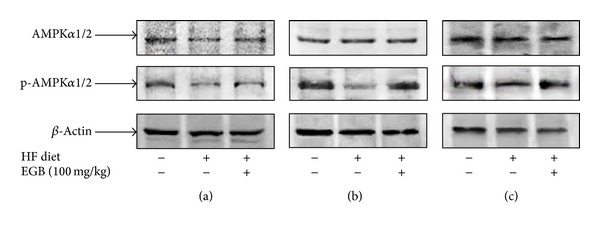
Effects of EGB on the expression of AMPK and p-AMPK in the liver (a), muscle (b), and fat (c) of HF diet rats. Each electrophoretogram is representative of the results from three individual experiments.

Because EGB effectively suppressed the development of impaired glucose tolerance, dyslipidemia, fatty liver, and endothelial dysfunction, the expression of AMPK was examined in liver, muscle, and fat tissues. The expression of AMPK was significantly decreased in HF diet group. However, treatment of EGB group increased expression levels of protein in liver, muscle, and fat tissues (Figure 7).

## 4. Discussion

Herb, Acupuncture, and Natural Medicine (HAN), one of the most ancient and revered forms of healing, has been used to diagnose, treat, and prevent disease for over 3,000 years. HAN is now used worldwide as an effective means of overcoming disease. *Gastrodia elata* is a well-known traditional Korean medicinal herb specifically for promoting blood circulation to remove blood stasis. In the present study, we provided the evidence for the beneficial effect of *Gastrodia elata* on lipid metabolism and endothelial dysfunction in high fructose-induced metabolic syndrome rat model.

Fructose is a lipogenic component, its consumption promotes the development of atherogenic lipid profile and elevation of postprandial hypertriglycemia [15, 16]. In addition, HF diet animals develop hypertriglyceridemia, obesity, impaired glucose tolerance, fatty liver, increased SBP, and vascular remodeling [17, 18]. In the present study, HF diet clearly increased visceral epididymal fat pads weight resulting from the increases in triglyceride and LDL cholesterol. Treatment with EGB lowered epididymal fat pads weight, triglyceride, and LDL cholesterol levels, whereas it elevated HDL cholesterol levels which assist lipid metabolism. Thus, EGB improves lipid metabolism by the decrease of triglyceride and LDL cholesterol. Although increased epididymal fat pads, body weight was not different from control diet and HF diet group. We suppose that proper experimental periods should be longer than the present periods for 8 weeks to increase body weight. It is sure that EGB is effective in obesity in HF diet rats, since EGB significantly decreased HF diet-induced increase in body weight.

In addition, disorder of lipid levels induced by HF diet was associated with aortic lesion. Histological analysis demonstrated that the endothelial layers were rougher in aortic sections of HF diet rats associated with a trend towards an increased development of atherosclerosis. Intima-media thickness of the thoracic aorta has been shown to correlate with prognosis and extend of coronary artery disease [19]. Treatment of EGB maintained smooth and soft intima endothelial layers and decreased intima-media thickness in aortic sections of HF diet rats.

Dyslipidemia, impaired glucose tolerance, and fatty liver are major features associated with metabolic syndrome in HF diet rats [19, 20]. Fructose induces impaired glucose tolerance via the elevation of plasma triglyceride levels. In addition, previous study demonstrated that an elevated fructose diet associated with impaired glucose tolerance and endothelial dysfunction precedes the development of hypertension [21]. Impaired glucose tolerance plays an important role in the development of such abnormalities as insulin resistance, type 2 diabetes, and dyslipidemia [22]. Similarly, HF diet induced impaired glucose tolerance and dyslipidemia, whereas treatment of EGB improved impaired glucose tolerance with the amelioration of dyslipidemia. In addition, EGB significantly suppressed the increasing adipocyte size and fatty liver. Thus, these results suggest that EGB may be useful to suppress the development of atherosclerotic lesions, obesity, and ameliorated lipid metabolism in metabolic syndrome model.

Endothelial dysfunction plays an important role in hypertension and vascular inflammation, other cardiovascular diseases, and metabolic syndrome [23, 24]. In this experimental model, the expression of ET-1 and inducible adhesion molecules such as ICAM-1, VCAM-1, and E-selectin in the arterial wall represent a key event in the development of atherosclerosis. EGB ameliorated vascular inflammation by downregulation of ET-1 as well as ICAM-1, VCAM-1, and E-selectin expressions in thoracic arota. Several studies have shown that lowering blood pressure and endothelial functions are related to an increase of eNOS reactivity, thereby increasing NO production roles as a strong vasodilator [25, 26]. In the present study, EGB upregulated eNOS levels in the aorta and recovered the HF diet-induced impairment of endothelium-dependent vasorelaxation. However, endothelium-independent vasodilator-induced vasorelaxation was not affected by EGB. These results suggest that hypotensive effect of EGB is mediated by endothelium-dependent NO/cGMP pathway. Histological study revealed that EGB suppressed vascular inflammation, compatible with the processes of atherosclerosis. In fact, endothelial dysfunction was initially identified as impaired vasodilation to specific stimuli such as ACh or bradykinin; therefore, improvement of endothelial function is predicted to regulate lipid homeostasis [27]. Thus, antihypertension and antivascular inflammatory effects of EGB contribute to the beneficial effects on endothelial function and lipid metabolism in metabolic syndrome.

To clarify the mechanism for EGB suppressing the development of visceral obesity, impaired glucose tolerance, dyslipidemia, and fatty liver, the study was focused on the expression of AMP-activated protein kinase (AMPK). There is a strong correlation between low activation state of AMPK with metabolic disorder associated with insulin resistance, fat deposition, and dyslipidemia [28–30]. AMPK is a key regulator of glucose and lipid metabolism. In the liver and muscle, activation of AMPK results in enhanced fatty acid oxidation and decreased production of glucose, cholesterol, and triglycerides [31]. Recently Misra reported that the suspected role of AMPK appeared as a promising tool to prevent and/or to treat metabolic disorders [32]. Also, the activation of AMPK signaling pathway is associated with eNOS regulation and alteration of systemic endothelin pathway in fructose diet animal models [25]. AMPK is required for adiponectin-, thrombin-, and histamine-induced eNOS phosphorylation and subsequent NO production in endothelium [33]. However, our study showed that EGB induced markedly not only activation of phosphorylation AMPK*α* in the liver, muscle, and fat, but also activation of eNOS levels in aorta. It could be hypothesized that EGB could lead to novel AMPK-mediated eNOS pathways which could in turn recover HF diet-induced metabolic disorders.

## 5. Conclusion

These results suppose that EGB ameliorates lipid metabolism, impaired glucose tolerance, hypertension, and endothelial dysfunction in HF diet-induced metabolic syndrome, at least in part, via activation of AMPK and eNOS/NO pathway. Therefore, *Gastrodia elata* Blume might be a beneficial therapeutic approach for metabolic syndrome.

## Figures and Tables

**Table 1:** Effect of EGB on body weight, epididymal fat pads, and blood glucose.

Groups	Control	HF	HF + EGB
Initial BW (g)	245.8 ± 7.6	244.4 ± 7.4	244.4 ± 9.0
Terminal BW (g)	449.4 ± 28.9	439.8 ± 26.5	402.5 ± 22.1^#^
Epididymal fat pads weight (g)	2.5 ± 0.7	3.9 ± 1.2**	2.5 ± 0.5^##^
Blood glucose (mg/dL)	94.63 ± 6.48	99.50 ± 7.30	96.70 ± 8.54

Values were expressed as mean ± SD (*n* = 10). **P < 0.01 versus Cont.; ^#^ *P* < 0.05, ^##^ *P* < 0.01 versus HF. HF: high fructose; HF + EGB: high fructose diet with EGB; BW: body weight.

**Table 2:** Effect of EGB on plasma lipid levels.

Groups	Control	HF	HF + EGB
T-Cho (mg/dL)	67.86 ± 7.6	102.94 ± 19.7**	67.79 ± 5.8^##^
TG (mg/dL)	83.83 ± 16.4	272.67 ± 107.0**	177.33 ± 59.6^#^
HDL-c (mg/dL)	13.75 ± 1.3	16.02 ± 2.9	20.2 ± 2.2^#^
LDL-c (mg/dL)	28.37 ± 3.9	44.56 ± 8.1**	24.28 ± 3.1^##^

Values were expressed as mean ± SD (*n* = 10). **P < 0.01 versus Cont.; ^#^ *P* < 0.05, ^##^ *P* < 0.01 versus HF. HF: high fructose; HF + EGB: high-fructose diet with EGB; T-Cho: total cholesterol; TG: triglyceride; HDL-c: high-density lipoprotein cholesterol; LDL-c: low-density lipoprotein cholesterol.
